# Salamander-Derived, Human-Optimized nAG Protein Suppresses Collagen Synthesis and Increases Collagen Degradation in Primary Human Fibroblasts

**DOI:** 10.1155/2013/384091

**Published:** 2013-10-31

**Authors:** Mohammad M. Al-Qattan, Medhat K. Shier, Mervat M. Abd-AlWahed, Ola H. Mawlana, Mohammed S. El-Wetidy, Reginald S. Bagayawa, Hebatallah H. Ali, May S. Al-Nbaheen, Abdullah M. Aldahmash

**Affiliations:** ^1^Department of Surgery, King Saud University, Riyadh, Saudi Arabia; ^2^College of Medicine Research Center, King Saud University, Riyadh, Saudi Arabia; ^3^Stem Cell Unit, Department of Anatomy, College of Medicine, King Saud University, Riyadh, Saudi Arabia; ^4^Endocrine Research Laboratory (KMEB), Odense University Hospital & University of Southern Denmark, Odense, Denmark

## Abstract

Unlike humans, salamanders regrow their amputated limbs. Regeneration depends on the presence of regenerating axons which upregulate the expression of newt anterior gradient (nAG) protein. We had the hypothesis that nAG might have an inhibitory effect on collagen production since excessive collagen production results in scarring, which is a major enemy to regeneration. *nAG* gene was designed, synthesized, and cloned. The cloned vector was then transfected into primary human fibroblasts. The results showed that the expression of nAG protein in primary human fibroblast cells suppresses the expression of collagen I and III, with or without TGF-**β**1 stimulation. This suppression is due to a dual effect of nAG both by decreasing collagen synthesis and by increasing collagen degradation. Furthermore, nAG had an inhibitory effect on proliferation of transfected fibroblasts. It was concluded that nAG suppresses collagen through multiple effects.

## 1. Introduction

Tissue regeneration and wound healing are two different processes [1]. In regeneration, there is a suitable microenvironment for new tissue formation. Human liver, spleen, and skeletal muscle retain some ability to regenerate after damage [2, 3]. Most of the new research in the field of regenerative medicine investigates the concept of stem cell-based tissue engineering approach for tissue regeneration [4, 5]. In contrast, wound healing is a process of tissue repair mainly by fibrous tissue (collagen) formation [6]. Although healing by fibrous tissue is essential for “normal” tissue repair, excessive fibrosis is a harmful pathological process. Examples of pathological fibrotic conditions in humans include hypertrophic/keloid scars [7], Dupuytren contracture [8], idiopathic pulmonary fibrosis [9], myocardial interstitial fibrosis [10], and hepatic fibrosis [11].

Regrowth of amputated limbs of salamanders represents a unique example of tissue regeneration because there is regeneration of different types of tissues in the correct orientation. Therefore, the molecular events of the regenerating amputation stump are of interest in the field of biotechnology. The amputation stump of the salamander forms a blastema which is a mound of proliferating mesenchymal cells surrounded by wound epithelium. Regeneration depends on the presence of regenerating axons which upregulate the expression of newt anterior gradient (nAG) protein [12–15]. nAG protein expression by schwann cells of regenerating axons peaks at 5–7 days postamputation. The nAG protein is released by schwann cells. At 10–12 days, the protein is also expressed in glands in the dermis underlying the wound epithelium [12–15]. The effect of nAG on dermal fibroblasts has not been previously investigated. We had the hypothesis that nAG might have an inhibitory effect of collagen production since excessive collagen production results in scarring, which is a major enemy to regeneration. The following experiments were conducted to test this hypothesis. We show that nAG suppresses collagen, both by decreasing collagen production and by increasing collagen degradation. We also show that nAG had an inhibitory effect on proliferation of transfected fibroblasts.

## 2. Materials and Methods

### 2.1. Ethics and Consent Statements

Primary human fibroblasts were isolated from infant human foreskin after obtaining written consent from the parents. Subjects (including human material or human data) reported in the current paper have been performed with the approval of the “College of Medicine Research Center, Deanship of Scientific Research, King Saud University, Riyadh, Saudi Arabia” Ethics Committee, in compliance with the Helsinki Declaration (http://www.wma.net/en/30publications/10policies/b3/index.html).

### 2.2. *nAG* Gene Design and Synthesis

**Figure 1:**
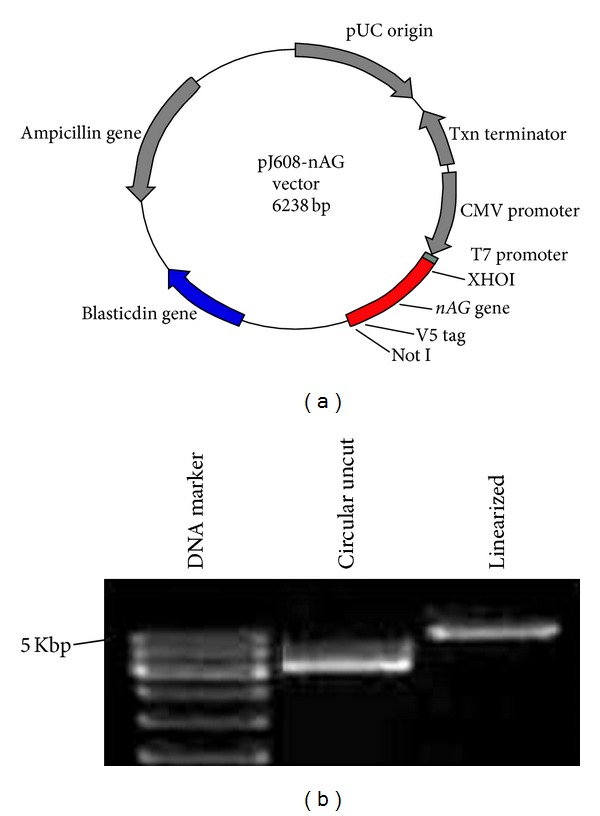
Identification of *nAG* gene in mammalian expression plasmid nAG-pJexpress pJ608. (a) The plasmid contains *nAG *gene, optimized for human cell expression and tagged with V5 peptide for further identification. The gene is surrounded by two restriction sites: XhoI and Not I. (b) nAG plasmid integrity was confirmed by DNA electrophoresis showing the plasmid in two forms: circular uncut plasmid and linearized cut with XhoI.

nAG mRNA and protein sequences were obtained from NCBI nucleotide database. *Notophthalmus viridescens* anterior gradient protein (eastern newt) 2 mRNA is 820 bases and the protein is 166 amino acids. The first amino acid methionine (ATG) starts at nucleotide 66 of the mRNA and the last amino acid leucine (CTG) ends at nucleotide 563 of the mRNA sequence. This was confirmed by using translate utility of the bioinformatics tools available from DNA 2.0 company online. nAG amino acid sequence (166 AA) was entered into DNA 2.0 Gene Designer Software. The DNA sequence was depicted automatically by the software, giving different options of codon usage for many amino acids. Adjusting the software to *homosapiens* (25% threshold), protein sequence is codon-optimized for efficient expression in human cells. Many amino acids are needed to be optimized for expression in human cells (more than 79%) (see Supplementary Material available online at http://dx.doi.org/10.1155/2013/384091). Eukaryotic regulatory elements including transcription elements (enhancer, promoter, and polyadenylation signal sequence) and replication elements were not included in the design of *nAG* gene. The only elements that were added are nAG mRNA 5′ UTR and V5 peptide for later detection of the protein using anti-V5 antibody. Transcription regulatory elements are supplied in the pJexpress 608 mammalian expression vector of DNA 2.0 Company (Figure 1(a)). *nAG* gene was also designed to contain two unique restriction enzymes Xho1 and Not 1, for further use in molecular cloning. Xho1 is designed to be upstream of the gene sequence and Not 1 to be downstream. Regarding GC content, Gene Designer software proposes only sequences with GC% between 30% and 70%. After optimization, the GC% of the *nAG *gene was 53.7%. nAG plasmid integrity was confirmed by DNA electrophoresis showing the plasmid (6.2 kbp) in two forms: circular uncut plasmid and linearized cut with XhoI (Figure 1(b)).

### 2.3. Transformation and Preservation of nAG-pJexpress 608

Confirmation of nAG-pJexpress 608 (synthesized by DNA 2.0, USA) was done by performing 1% agarose gel electrophoresis for both circular plasmid and digested plasmid with Xho1 enzyme (Figure 1). Propagation of plasmid was performed by transformation of nAG plasmid into competent DH5*α* bacterial cells, prepared in our laboratory by using CaCL_2_ method according to the protocol of Current Protocols in Molecular Biology. Transformation was done with 10 ng of nAG-pJexpress 608 added to 100 *μ*L of competent DH5*α* heat shocked for 45 sec at 42°C followed by incubation on ice for 10 minutes, then selection of transformed bacterial cells on Ampicillin LB agar plates, and finally preservation of bacterial cells in glycerol stock at −80°C.

### 2.4. Fibroblasts Isolation and Culture

Dermal specimen was washed repeatedly with phosphate-buffered saline (PBS) (Gibco, Carlsbad, California, USA) with a combination of 1% penicillin and streptomycin sulfate (Gibco) and minced aseptically into approximately 1 mm^3^ pieces. The specimens were then placed in 10 cm culture plate with 5 mL of culture medium (Dulbecco Modified Eagle Medium (DMEM), 10% fetal bovine serum, 1% nonessential amino acid, and 1% penicillin-streptomycin sulfate) (Gibco) at 37°C in a humidified 5% CO_2_ incubator. After 5 days the medium was repeatedly changed every 48 h and examined under microscope untill fibroblasts were seen growing outwards from the explanted tissue. Then the tissue was removed and sufficient outgrowth of fibroblasts was subcultured and propagated. For testing collagen synthesis experiments, 150 *μ*g/mL L-ascorbic acid (Bio Basic Inc., Ontario, Canada) and TGF-*β*1 (Santa Cruz, Texas, USA) 3 ng/mL (for collagen I) and 10 ng/mL (for collagen III) were added for culture medium.

### 2.5. Plasmid Transfection (Lipofection and Electroporation) into Primary Human Fibroblast

Lipofection for nAG plasmid was carried out using Lipofectamine 2000 (Invitrogen, Carlsbad, California, USA); cells were seeded in a complete medium without antibiotic until they reach 80–90% confluence in 6 cm plates. Lipofection was done using 1 : 3 DNA : Lipofectamine ratio. DNA : Lipofectamine complexes were removed after 5 hours and replaced with complete medium.

Electroporation for nAG plasmid was performed into primary human fibroblasts by aliquoting 800 *μ*L of cell suspension in electroporation buffer (150 mOsmol/kg) and 20 *μ*g of nAG plasmid into a cuvette with 4 mm gap width. Multiporator (Eppendorf, Hamburg, Germany) was adjusted on eukaryotes mode, 800 V with time constant 60 *μ*sec and the cells received two pulses. After 10 min, the cells were plated in 6 cm plates for western blot assay and in 8 chambers slide (Millipore, Billerica, MA, USA) for immunofluorescence assay.

### 2.6. Western Blotting

After 24 h and 48 h of transfection, cells were scrabed and lysed using RIPA cocktail (RIPA buffer, PMS, sodium orthovanadate, and protease inhibitor) (Santa Cruz). Cell suspensions were centrifuged at 14.000 rpm for 15 minutes at 4°C. Total cellular protein concentrations were measured by Bradford reagent (Sigma, Louis, USA). Equal amounts of proteins were separated on SDS polyacrylamide gel 12% for nAG separation (18.9 kd) and 7.5% SDS polyacrylamide gel for collagen I (90 kd) and collagen III (140 kd) at 100 V for 2 h. Proteins were transferred into PVDF membranes (Millipore) using 100 V for 1.5 h. After the transfer, membranes were blocked by 10% blocking buffer Blotto (Santa Cruz) dissolved in TBST for 1 hr at room temperature. The membranes then were incubated with primary antibodies (V5-probe, Santa Cruz) dissolved in blocking buffer (1 : 600) for nAG detection, (COL1A1, Santa Cruz) (1 : 200) for collagen I detection, (COL3A1, Santa Cruz) (1 : 200) for collagen III detection, and *β*-actin (sc-69879, Santa Cruz) (1 : 1000). After wash, the membranes were incubated with the appropriate HRP-conjugated secondary antibody (Santa Cruz) dissolved in blocking buffer (1 : 10000) for 1 h at room temperature. After washing the immunoblots were visualized by ECL kit (Amersham, Buckinghamshire, UK) and the bands' densities were quantified using Quantity One software (Bio-Rad). Results were expressed as ratio of band density to *β*-actin.

### 2.7. Immunofluorescence

Transfected cells were plated at density of 8 × 10^4^ cells/well, and after 48 hours, medium was removed and cells were fixed and permeabilized by 2% PFA (Santa Cruz)/0.1% Triton x-100 (LKB Bromma, Sweden) in PBS for 30 minutes. After washing, cells were blocked by 1% BSA (Santa Cruz) in PBST (1% Triton X-100 in PBS) for 30 minutes. The cells were incubated with primary antibodies V5-probe (sc-58052, Santa Cruz) for nAG detection, COL1A1 (sc-28657, Santa Cruz) for collagen I detection, and COL3A1 (sc-8780-R, Santa Cruz) for collagen III detection using 1 : 50 dilution in blocking buffer for 1 hour at room temperature. After washing, bound primary antibodies were detected by incubation with the appropriate FITC-conjugated secondary antibodies (Santa Cruz) 1 : 100 dilution for 1 hour in a dark chamber and then examined under fluorescence microscope (Olympus, Center Valley, PA, USA).

### 2.8. Proliferation Activity of Nontransfected and nAG Transfected Fibroblasts (BrdU Incorporation Assay)

The cells were cultured in 96-well plates at a density of 8000 cells/well in complete growth media. After 24 and 48 hours, proliferation of non-transfected and nAG transfected fibroblasts was assayed by incorporation of pyrimidine analogue 5-bromo-2′-deoxyuridine (BrdU) using ELISA, BrdU (Roche, Mannheim, Germany). Cells were labeled using 10 *μ*M BrdU per well and incubated for 2 h at 37°C in a humidified atmosphere. After fixation, the cells were incubated with the anti-BrdU-POD antibody for 90 minutes at room temperature. After the removal of the antibody conjugate, the cells were washed and the substrate solution was added. The reaction product was quantified by measuring the absorbance using a Microplate Reader Synergy 2 (BioTek, Winooski, Vermont, USA) at 370 nm.

### 2.9. Collagen Synthesis

#### 2.9.1. RNA Extraction and Quantitative Real-Time PCR Analysis

Total RNA was isolated both from fibroblasts transfected with nAG plasmid and from normal fibroblasts by using the RNeasy protect mini kit (Qiagen, Dusseldorf, Germany) according to the manufacturer's instructions. 100 ng of total RNA was reverse-transcribed and target genes expression was measured in multiplex, one-step RT-PCR using rotor-gene multiplex PCR kit (Qiagen, Dusseldorf, Germany) according to the manufacturer's instructions. Specific primers and TaqMan probes were designed using Integrated DNA Technologies software (San Diego, California, USA). The TaqMan probes had a fluorescent reporter dye (FAM, HEX, or ROX) attached to the 5′ end, while the Quencher BHQ1 (for FAM and HEX) and BHQ2 (for ROX) were attached to the 3′ end. Sequences of the primers and probes used are listed in Table 1. Thermal cycling conditions were as follows: an initial reverse transcription step for 15 min at 50°C, incubation at 95°C for 5 min to activate hot start DNA polymerase, and then 40 cycles at 95°C for 15 sec and 60°C for 15 sec. Acquiring of the fluorescent signal on green, yellow, and orange channels occurs in the annealing/extension step. Using *β*-actin as a normalizing gene and procollagen I and procollagen III in normal fibroblasts as calibrators, relative quantification of procollagen I and procollagen III expression levels in nAG transfected fibroblasts was obtained using ΔΔct relative quantification method in Rotor-gene Q 5plex software (Qiagen). The relative mRNA expressions in transfected and control fibroblasts were determined by three independent quantitative real-time PCR experiments.

### 2.10. Collagen Degradation

#### 2.10.1. Gelatin Zymography for MMP-2 Detection

MMP proteins with gelatinolytic activity were determined in conditioned media deprived from serum for 24 hours for normal fibroblasts and fibroblasts transfected with nAG plasmid and treated with 3 ng/mL TGF-*β*1. 20 uL of undiluted cell culture supernatant was mixed with an equal volume of nonreducing SDS sample buffer, using MMP-2 (R&D Systems, Minneapolis, USA) as positive control. Samples were loaded on 7.5% separating SDS polyacrylamide gel with 0.1% gelatin (LOBA Chemie, Mumbai, India) as substrate and electrophoresis was done at constant voltage of 100 V for 2 hours. The gels were then washed in renaturing buffer (2.5% Triton X-100) for 1 hour to promote recovery of protease activity before incubation in zymogram development buffer at 37°C. The gels were stained with Coomassie blue R-250. After destaining, clear digested regions representing MMP gelatinolytic action appeared against blue background. The optical density and area of the bands were compared using densitometry in gel documentation instrument (Bio-Rad, Berkeley, USA).

#### 2.10.2. Assessment of Pro-MMP-1 Level Using ELISA

ELISA was performed using Quantikine Human Pro-MMP-1 ELISA (R&D Systems, Minneapolis, USA). Protocol was done as per the manufacturer directions. Standards and samples (culture media of non transfected fibroblasts, fibroblasts transfected with nAG plasmid, and fibroblasts treated with 1 *μ*M recombinant nAG) were added and incubated in microplate wells precoated with anti-pro-MMP-1 mouse monoclonal antibody. After washing away unbound substances, horseradish peroxidase linked monoclonal antibody specific for pro-MMP-1 is added to the wells. Following a wash and addition of substrate solution, the reaction was stopped and the resultant colour was read at 450 nm using microplate reader Synergy 2 (BioTek, Winooski, Vermont, USA).

### 2.11. Statistical Analysis

Data were expressed as means ± standard deviation (SD) and analyzed by one-way ANOVA test and *P* value less than 0.05 was considered statistically significant. The statistical difference was determined using Student' *t*-test for independent groups. All experiments were performed at least three replicates.

## 3. Results

### 3.1. nAG Expression in Primary Fibroblast Cells

**Figure 2:**
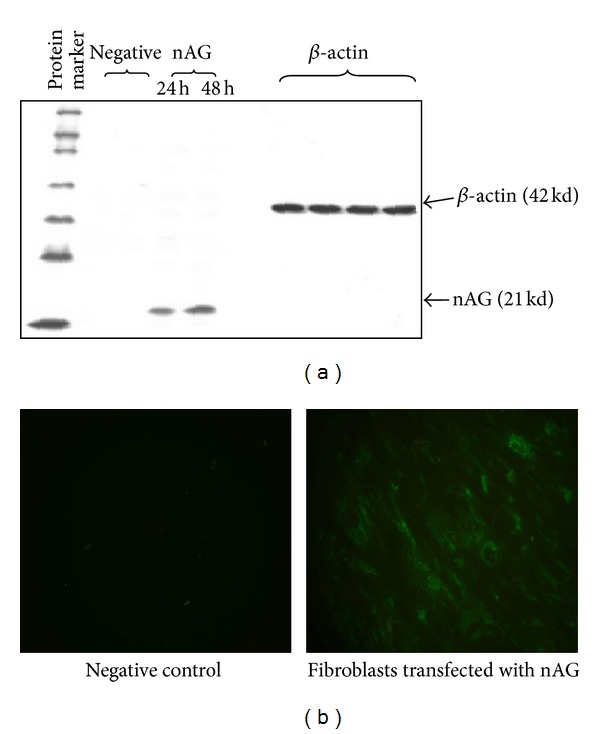
Western blot assay (a) and immunofluorescence (b) experiments showing nAG protein expression in primary human fibroblasts. (a) Western blot was performed after 24 h and 48 h of transfection. The cells were lysed by using RIPA cocktail; proteins were separated on 12% SDS polyacrylamide gel. The primary antibody V5 probe and HRP-conjugated secondary antibody were used for nAG protein detection in the following lanes: nontransfected fibroblasts (negative control 1), fibroblasts with nAG plasmid without lipofection (negative control 2), nAG transfected fibroblasts tested after 24 h and nAG transfected fibroblasts tested after 48 h. (b) Immunofluorescence assay was performed after 48 h of transfection and cells were fixed and permeabilized by 2% PFA/0.1% Triton x-100. V5 probe was used as primary antibody and green fluorescence was detected by using FITC-conjugated secondary antibody. Compare nAG protein expression in nontransfected fibroblasts (negative control) to nAG expression in transfected fibroblasts (magnification 40x).

Western blot assay confirmed nAG protein expression in human fibroblasts showing that a specific nAG protein band corresponds to 18.9 kd, nAG protein expression appeared after 24 hours, and the amount of protein expression increased after 48 hours as shown in the increasing band density (Figure 2(a)).

Immunofluorescence staining with primary antibody V5-probe showed nAG protein expression in primary human fibroblast and fluorescent green color was evident after 24 hours of pJ608-nAG plasmid transfection (Figure 2(b)).

### 3.2. Proliferation Activity in Nontransfected and nAG Transfected Fibroblasts

**Figure 3:**
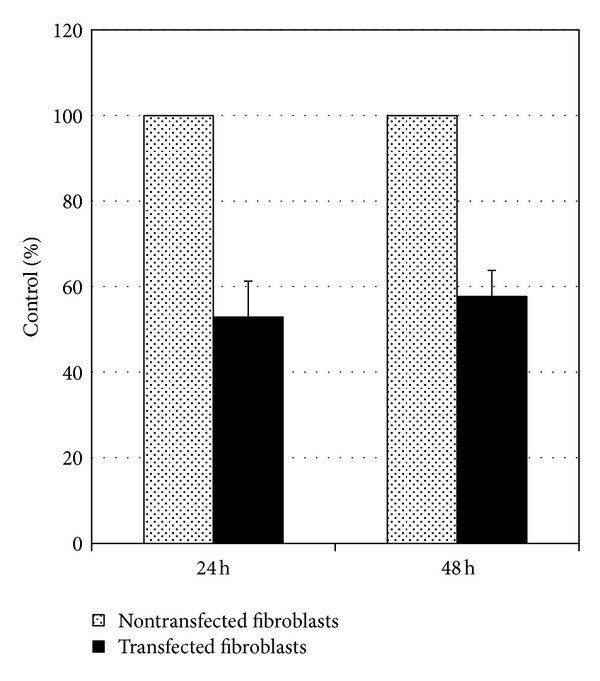
BrdU incorporation ELISA assay for assessment of proliferation activity in nontransfected and nAG transfected fibroblasts. The cells were cultured in 96-well plates at a density of 8000 cells/well. 24 and 48 hours after lipofection, cells were assayed for proliferation by measuring BrdU incorporation during DNA synthesis in proliferating cells. The results showed the inhibitory effect of nAG on fibroblasts proliferation, after 24 h by 47% decrease (*P* < 0.0001) and after 48 h by 42% decrease (*P* < 0.0001) in proliferation in nAG transfected fibroblasts compared to nontransfected fibroblasts.

BrdU incorporation ELISA assay was performed to test effect of nAG on fibroblasts' proliferation. The results showed the inhibitory effect of nAG on fibroblasts proliferation. Compared to control (non-transfected fibroblasts), there was inhibition in proliferation by 47% decrease (SD ± 8.39) (*P* < 0.0001) after 24 h of transfection and by 42% decrease (SD ± 6.096) (*P* < 0.0001) after 48 h in nAG transfected fibroblasts (Figure 3).

### 3.3. Collagen Expression in Fibroblasts Expressing nAG with or without TGF-*β*1

**Figure 4:**
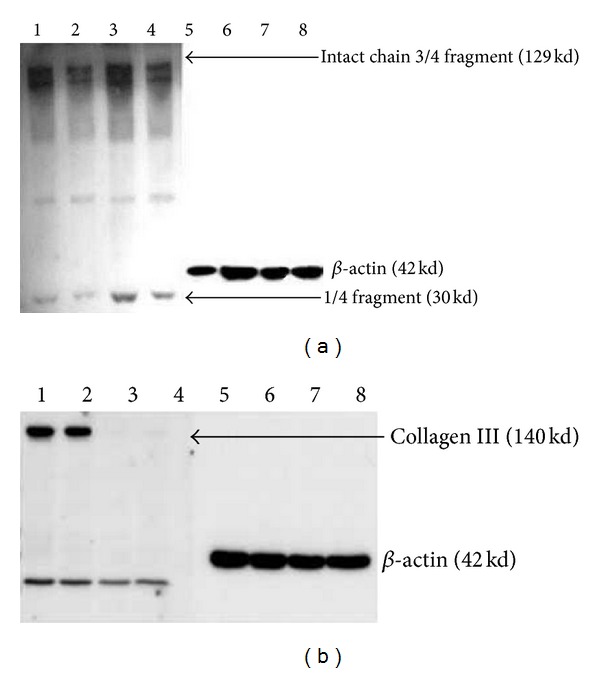
Western blot experiment for detection of collagen I (a) and collagen III (b) expression in primary human fibroblasts transfected with nAG plasmid with or without treatment with TGF-*β*1. Cells were lysed after 48 hours using RIPA cocktail. Proteins were separated on 7.5% SDS polyacrylamide gel and detected by using COL1A1 (primary antibody for collagen I), COL3A1 (primary antibody for collagen III), and HRP-conjugated secondary antibody and the immunoblots were visualized by using ECL kit. (a) Collagen I detection: lane-1: collagen I expression in non-transfected fibroblasts without any treatment (control 1), lane-2: collagen I expression in fibroblasts transfected with nAG plasmid, lane-3: collagen I expression in non-transfected fibroblasts treated with TGF-*β*1 (control 2), lane-4: collagen I expression in fibroblasts transfected with nAG plasmid and treated with TGF-*β*1, and lanes-5–8: internal control *β*-actin. (b) Collagen III detection: lane-1: collagen III expression in non-transfected fibroblasts without any treatment (control 1), lane-2: collagen III expression in non-transfected fibroblasts with TGF-*β*1 treatment (control 2), lane-3: collagen III expression in fibroblasts transfected with nAG plasmid, lane-4: collagen III expression in fibroblasts transfected with nAG plasmid and treated with TGF-*β*1, and lanes-5 to 8 are internal control *β*-actin.

Western blot assay showed collagen I expression in untreated control fibroblasts (Lane 1, Figure 4(a)) and control fibroblasts treated with TGF-*β*1 (Lane 3, Figure 4(a)). Transfected fibroblasts without treatment with TGF-*β*1 (Lane 2, Figure 4(a)) showed decreased collagen I expression compared to untreated control fibroblasts. With TGF-*β*1 treatment, there was also decreased collagen I expression in transfected fibroblasts (Lane 4, Figure 4(a)) compared to treated control fibroblasts. The effect on collagen I involved both full length protein (129 KD) and the degradation fragment (30 KD) (Figure 4(a)).

Western blot assay showed collagen III expression in untreated control fibroblasts (Lane 1, Figure 4(b)) and control fibroblasts treated with TGF-*β*1 (Lane 2, Figure 4(b)). Transfected fibroblasts without treatment with TGF-*β*1 (Lane 3, Figure 4(b)) showed decreased collagen III expression compared to untreated control fibroblasts. With TGF-*β*1 treatment, there was also decreased collagen III expression in transfected fibroblasts (Lane 4, Figure 4(b)) compared to treated control fibroblasts. The effect on collagen III was complete suppression of the full length protein (140 KD). This indicated that nAG protein expression in human fibroblast cells has an inhibitory effect on the expression of collagen I and III and that the inhibitory effect of nAG is dominant over the TGF-*β*1 effect. Furthermore, the inhibitory effect of nAG protein was more pronounced on collagen III than collagen I.

**Figure 5:**
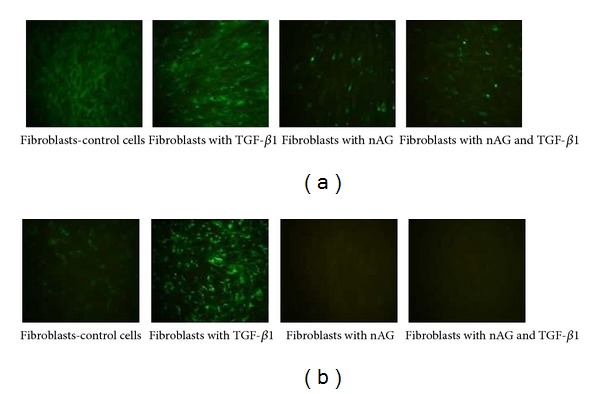
Immunofluorescence experiment showing effect of nAG on collagen I (a) and collagen III (b) expression in primary human fibroblasts with or without TGF-*β*1 treatment. After 48 h of transfection, immunoflourescence was performed by using COL1A1, COL3A1 (primary antibodies), and FITC-conjugated secondary antibody. (a) Immunofluorescence staining of type I collagen is in the following order: control fibroblasts (complete medium + 150 *μ*g/mL L-ascorbic acid), control fibroblasts treated with 3 ng/mL TGF-*β*1, fibroblasts with nAG plasmid, and fibroblasts with nAG plasmid and treated with 3 ng/mL TGF-*β*1 (magnification 40x). Note the suppressive effect of nAG on collagen I. (b) Immunofluorescence staining of type III collagen is in the following order: control fibroblasts (complete medium + 150 *μ*g/mL L-ascorbic acid), control fibroblasts treated with 10 ng/mL TGF-*β*1, fibroblasts with nAG plasmid, and fibroblasts with nAG plasmid and treated with 10 ng/mL TGF-*β*1 (magnification 40x). Note the complete suppressive effect of nAG on collagen III.

Immunofluorescence (Figure 5) confirmed the results of the western blot assay. There was decreased collagen I expression in transfected fibroblasts (with and without TGF-*β*1) compared to controls (Figure 5(a)); and there was complete collagen III suppression in transfected fibroblasts (with and without TGF-*β*1) compared to controls (Figure 5(b)). This also confirmed that the suppressive effect of nAG was more pronounced on collagen III expression than collagen I.

### 3.4. Quantitative Real-Time PCR Analysis

**Figure 6:**
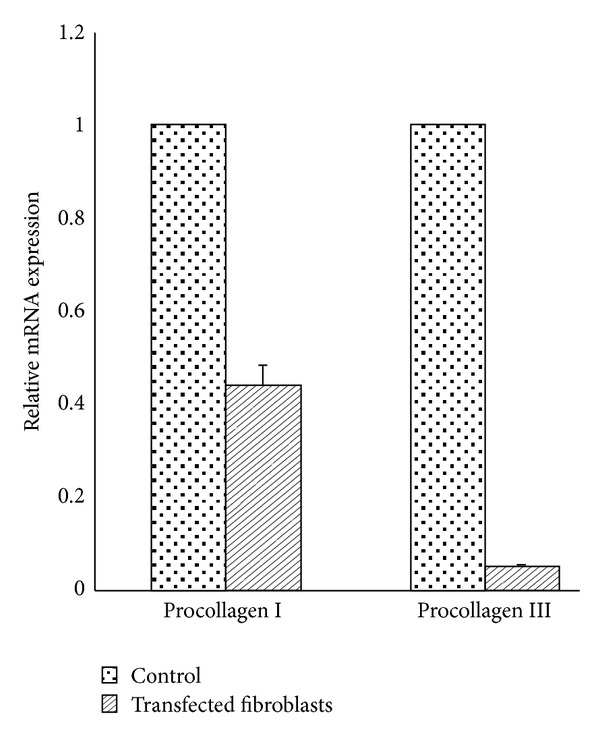
Quantitative real-time PCR (RT-PCR) measuring relative mRNA expressions level of procollagen I and procollagen III in non-transfected and nAG transfected primary human fibroblasts. 100 ng of total RNA was reverse-transcribed and target genes expression was measured in multiplex, one-step RT-PCR by using TaqMan probes with (FAM, HEX, or ROX) reporter dyes and (BHQ1 or BHQ2) quencher. To estimate effect of nAG on collagen I and collagen III synthesis, the relative mRNA expressions were related to the reference gene, *β*-actin. The relative expression of procollagen I was 55% decrease (*P* < 0.001) and procollagen III was 95% decrease (*P* < 0.0001) in nAG transfected fibroblasts compared to non-transfected fibroblasts. The data represents the mean of three independent experiments.

Relative quantification of mRNA expression levels in non-transfected and nAG transfected fibroblasts was performed to quantify the effect of nAG on procollagen I and procollagen III mRNA synthesis. The results confirmed that nAG protein suppresses procollagen I mRNA expression by 55% decrease (SD ± 0.028) (*P* < 0.001) and suppresses procollagen III mRNA expression by 95% decrease (SD ± 0.011) (*P* < 0.0001) in transfected fibroblasts than in non-transfected cells (Figure 6). The data represents the mean of three independent experiments.

### 3.5. Gelatin Zymography for MMP-2 Detection

**Figure 7:**
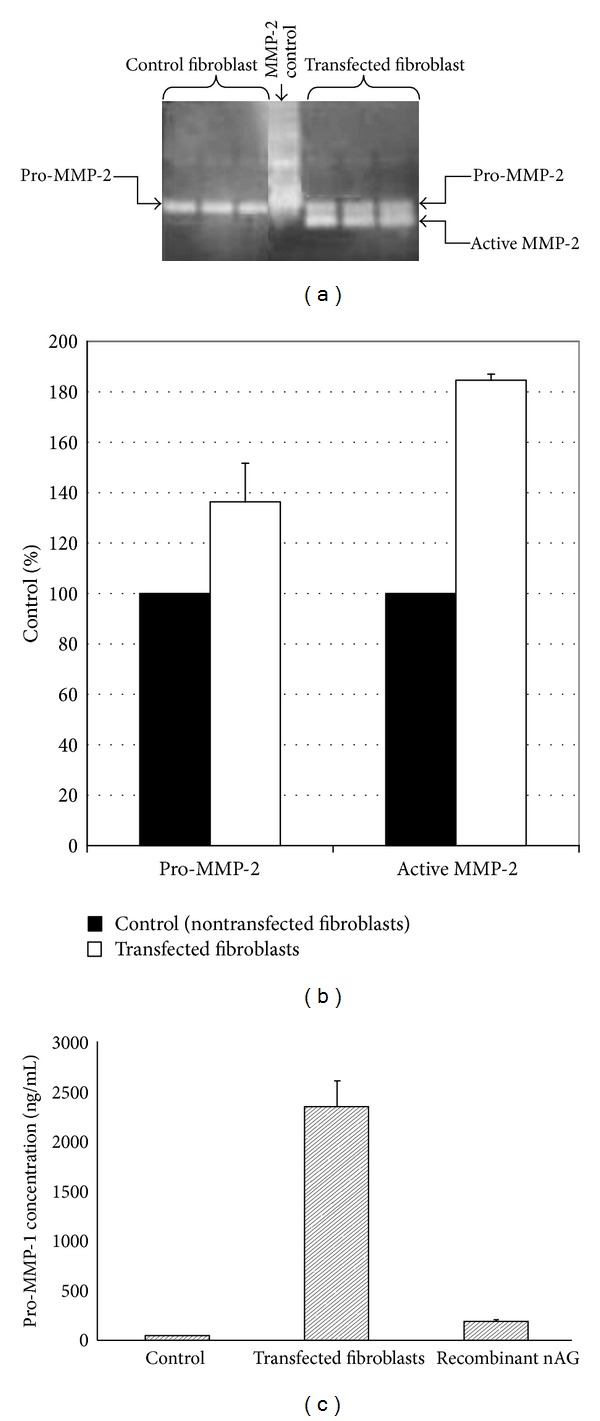
Determination of pro-MMP-2 and active MMP-2 by gelatin zymography ((a) and (b)) and pro-MMP-1 by ELISA (c) in nontransfected and nAG transfected fibroblasts. (a) Gelatin zymography: media were harvested after two days of transfection of nAG plasmid into fibroblasts and non-transfected fibroblasts (both were treated with 3 ng/mL TGF-*β*1). The first three lanes were different fractions of the same sample of non-transfected fibroblasts, the fourth lane was MMP-2 (positive control), and the last three lanes were samples of nAG transfected fibroblasts. (b) Densitometry analysis for gelatin zymography: density of each band was measured by using densitometry tool in Gel documentation software. There was 37% increase in pro-MMP-2 (*P* < 0.019) and 85% increase in active MMP-2 (*P* < 0.001) in transfected fibroblasts compared to non-transfected fibroblasts. (c) ELISA assay for pro-MMP-1 measurement: after two days of transfection. Media were harvested for measurement of pro-MMP-1 in non-transfected fibroblasts (control), nAG transfected fibroblasts, and fibroblasts treated with recombinant nAG. The level of pro-MMP-1 was 53-fold increase in transfected fibroblast compared to non-transfected fibroblasts (*P* < 0.004) and it was 4-fold increase in nAG-treated fibroblasts compared to non-transfected fibroblasts (*P* < 0.0001).

We performed gelatin zymography to determine the effect of nAG on MMP-2 gelatinase activity (which degrades gelatin and collagen) in non-transfected (control cells) and nAG transfected fibroblasts (Figure 7(a)). There were 37% increase in pro-MMP-2 (SD ± 15.33) (*P* = 0.019) and 85% increase in the active form of MMP-2 (SD ± 2.38) (*P* = 0.001) in nAG transfected fibroblasts compared to non-transfected fibroblasts (Figure 7(b)). This indicated that nAG increases collagen degradation. 

### 3.6. Pro-MMP-1 Level Using ELISA

Assessment of pro-MMP-1 protein level in culture media of non-transfected fibroblasts (control), nAG transfected fibroblasts, and fibroblasts treated with nAG recombinant protein showing that there was 53 fold increase in pro-MMP-1 expression levels (SD ± 257.9) (*P* = 0.004) in culture media of nAG transfected fibroblasts compared to non-transfected fibroblasts, and there was 4-fold increase in pro-MMP-1 expression levels (SD ± 18.03) (*P* < 0.0001) in fibroblasts treated with recombinant nAG compared to untreated cells (Figure 7(c)). Since MMP-1 collagenase activity has the ability to cleave the native helical structure of interstitial collagen I, II, and III, these results indicated that nAG increases collagen degradation.

## 4. Discussion

The current work opens new insights on the effects of nAG on collagen. We have designed a *nAG* gene that is suitable for human cells. We demonstrated the successful expression of nAG in human fibroblasts and the suppressive effect of nAG on the expression of collagen I and III. No previous studies have investigated the effect of nAG on collagen and hence we are unable to compare our results to others.

Although collagen formation is essential for tissue healing, excessive collagen is pathological [16]. The TGF-*β* pathway mediates both normal wound healing and scar formation [17]. Following the activation of the receptors by TGF-*β*1, SMADs 2 and 3 are phosphorylated and the activated SMADs bind to SMAD-4. This process is controlled by the inhibitory SMAD-7. The activated SMAD complex will then translocate from the cytoplasm into the nucleus to regulate gene expression for collagen synthesis and degradation [18–20]. 

Pathological fibrosis is characterized by abnormally high levels of TGF-*β*, increased proliferation of fibroblasts, increased collagen synthesis, decreased collagen degradation, and the appearance of myofibroblasts [21–25].

Type I collagen is the most abundant and is synthesized in response to injury [26]. Type III collagen is also synthesized in response to dermal injury. In normal wound healing, the newly formed type III collagen is gradually replaced by collagen I to maintain the normal collagen I to collagen III ratio in the dermis. In contrast, there is persistent expression of excessive type III collagen in pathological fibrosis [27]. 

Matrix metalloproteinase (MMPs) are considered to be the most important enzymes mediating collagen degradation [28]. The active forms of MMPs are formed when the prodomains are removed by autolytic cleavage or by other proteases [29, 30]. Normally, there is a balance between the MMPs and their inhibitors known as tissue inhibitors of matrix metalloproteinase (TIMPs). In pathological fibrosis this balance is disturbed with marked decrease in MMPs. The imbalance leads to increase collagen accumulation.

Our results showed that nAG has an inhibitory effect on collagen expression by both decrease in collagen production and increasing collagen degradation. This dual effect is interesting because pathological fibrosis is characterized by both increased collagen production and decreased collagen degradation. Another interesting finding is the fact that the suppressive effect of nAG on collagen is evident with or without TGF-*β*1 stimulation. This dominance over the TGF-*β*1 effect is important because excessive TGF-*β*1 stimulation is a feature of several fibrotic conditions [31]. Finally, our work demonstrated that the suppressive effect of nAG on collagen production is more pronounced on collagen III compared to collagen I. This is also interesting because collagen III overproduction is the hallmark of pathological fibrosis [32].

## 5. Conclusion

nAG suppresses collagen expression. This suppressive effect is mediated through multiple actions including decreased fibroblast proliferation, decreased collagen I and III production, and increased collagen degradation. Furthermore, the effects of nAG are dominant over TGF-*β* stimulation.

## Supplementary Material

Sequence alignment of the original nAG mRNA and the newly designed nAG.

## Figures and Tables

**Table 1:** Primers and probes used for quantitative real-time PCR.

Gene	Gene accession number	Primer, probe sequence	Product size
Procollagen I	AC number NM_000089.3	Forward: TGG ATT GAC CCT AAC CAA GGA TGC	145
		Reverse: AGA CGT GTT TCT TGT CCT TGG AGC	
		Probe: ACT GGC GAA ACC TGT ATC CGG GCC CAA CCT	

Procollagen III	AC number NM_000090.3	Forward: AGT CCT GGT GGT AAA GGC GAA ATG	150
		Reverse: TTT GGC ACC ATT CTT ACC AGG CTC	
		Probe: AAT GGT GCT CCT GGA CTG CGA GGT GGT GCA	

*β*-actin	AC number NM_001101.3	Forward: ACCGAGCGCGGCTACAG	60
		Reverse: CTTAATGTCACGCACGATTTCC	
		Probe: TTCACCACCACGGCCGAGC	
