# Supplementary material for: Salamander-Derived, Human-Optimized nAG Protein Suppresses Collagen Synthesis and Increases Collagen Degradation in Primary Human Fibroblasts
Source: Biomed Res Int. 2013 Oct 31;2013:384091. doi: 10.1155/2013/384091 (PMC3833026; doi:10.1155/2013/384091)
Supplement: Supplementary file 1 — Sequence alignment of the original nAG mRNA and the newly designed nAG. [file 384091.f1.docx]

**Supplementary Figure: Sequence alignment of original nAG mRNA and newly designed nAG.**

5'UTR

CACGAGTGGAGCACTTCCCCAGCACGCAAGACAAGTGCAGGTGGGAGACCAAGC CATCGCTCAAC…………………………………………...........Original

XHO1 (restriction site)

5'UTR

CTCGAGCACGAGTGGAGCACTTCCCCAGCACGCAAGACAAGTGCAGGTGGGAGACCAAGC CATCGCTCAAC……………………………..………….………Optimized

1 * ATG GTG AAA GGT TAC CTG GCA GCT CTT CTG CTC CTA GCG CTT TCT TCA TTC

1 † ATG GTG AAA GGG TAT TTG GCG GCG TTG CTT CTC CTT GCG CTG AGC TCA TTC

1 # **M V K G Y L A A L L L L A L S S F**

52 * AGC CTA GCC AAA GAG AGC GCC AAG AGA CCG GAA GTG AAG AAG GTC CAG ACT

52 † TCC CTC GCC AAA GAG TCG GCA AAG AGG CCC GAG GTG AAA AAG GTA CAG ACC

18 # **S L A K E S A K R P E V K K V Q T**

103 * CTT TCG AGG GGG TGG GGC GAC AGT CTC GAA TGG GCT CAG ACG TAT GAG GAA

103 † CTC TCG CGC GGT TGG GGA GAT AGC CTG GAA TGG GCG CAA ACG TAT GAA GAG

35 #  **L S R G W G D S L E W A Q T Y E E**

154 * AGC CTG TCC AAA TCC AGG AGC AGC AAC AAA CCA CTG CTC GTT ATC AAC CAC

154 † TCA TTG TCC AAG TCG AGA TCA TCG AAC AAA CCC CTG CTG GTG ATC AAT CAC

52 # **S L S K S R S S N K P L L V I N H**

205 * AGA GAT GAC TGT CCA CAC TCT CAA GCT TTG AAG AAA GCA TTT GCT GAG CAC

205 † CGC GAT GAC TGC CCT CAT TCG CAA GCT TTG AAG AAA GCA TTC GCG GAG CAC

68 # **R D D C P H S Q A L K K A F A E H**

256 * AAA GGC ATC CAG AAA CTC GCA GAG AAG TTC ATT CTT CTT AAC GTT GTT CAT

256 † AAG GGG ATC CAG AAG TTG GCC GAG AAG TTT ATC TTG CTC AAC GTG GTA CAC

85 # **K G I Q K L A E K F I L L N V V H**

307 * GAT CCA ACT GAC AAG AAC CTT GTA CTT GAT GGC ATG TAT GTA CCC AAG CTT

307 † GAT CCC ACC GAT AAG AAT CTG GTC TTG GAT GGG ATG TAC GTA CCA AAA CTC

102 # **D P T D K N L V L D G M Y V P K L**

358 * GTT TTC GTA GAT CCA TCT ATG GTA GTG AGA GCT GAT CTT CCT GGA AAA TAC

358 † GTC TTT GTG GAC CCT AGC ATG GTC GTC AGG GCC GAC CTC CCG GGA AAG TAC

119 # **V F V D P S M V V R A D L P G K Y**

409 * TCC AAT CAT CGG TAC ACC TAT GAG CCT GCA GAC ATT GAT CTG TTG TAT GGT

409 † TCG AAT CAT CGG TAC ACT TAC GAA CCC GCG GAC ATT GAC CTT CTC TAT GGC

136 # **S N H R Y T Y E P A D I D L L Y G**

460 * AAC ATG CAG AAA GCA CTC AAA CTT CTG AAA ACT GAA CTG **…… V5 Peptide……**

460 † AAT ATG CAG AAA GCA CTT AAG TTG CTG AAA ACG GAG CTT GGA AAA CCG ATT

153 # **N M Q K A L K L L K T E L G K P I**

**……………………………… V5 Peptide………………………………………**

511 † CCG AAC CCA CTC CTT GGT CTG GAC TCC ACA TGA GCGGCCGC

170 # **P N P L L G L D S T ***

**Not1 (restriction site)**

3'UTR

TGAGCGAAGAATGCCTAGACAAGTGACCCCCGCATCCTGTTTCCGCATGAGACTGCACAACCAGAAAGTTGACTTCAGTTGATTTGAAATTCATGAAGACACTGTAAAAGCATAACTGGGATTATGATTCATCTGGCTGTAAACACTTCCTGGCATTTTGACGTTTGACTGTGCTAGATTTTTTTAAAATGTATTCTTTATGCTTCATCTGTAAGCAACACATTTTT AAATAAATCCATTTTTGGGTATTTATTATT……………………………..………..original

* Original sequence

† Optimized sequence

# Amino Acids sequence
